# Becoming a (Neuro)migrant: Haitian Migration, Translation and Subjectivation in Santiago, Chile

**DOI:** 10.1080/01459740.2024.2324890

**Published:** 2024-03-06

**Authors:** Gabriel Abarca-Brown

**Affiliations:** Center for Culture and the Mind (CULTMIND), University of Copenhagen, Copenhagen, Denmark

**Keywords:** Chile, Haitian migration, psy technologies, translation, neurobiology, moral frameworks, Chile, Migración haitiana, Tecnologías Psi, Traducción, Neurobiología, Marcos Morales

## Abstract

Based on a multi-sited ethnography conducted over 14 months in northern Santiago, I examine how the introduction of a series of health policies and the global mental health agenda has interacted with and impacted Haitian migrants in the context of a postdictatorship neoliberal Chile (1990–2019). Specifically, I explore the interactions between health and social institutions, mental health practitioners, psy technologies, and Haitian migrants, highlighting migrants’ subjectivation processes and everyday life. I argue that Haitian migrants engage with heterogeneous subjectivation processes in their interactions with health and social institutions, challenging normative values of integration into Chilean society. These processes are marked not only by the presence of, or exposure to, psy interventions and mental health discourses but also by the degree of compatibility between a psychiatric and neurological language and Haitians’ ideals and moral frameworks.

## RESUMEN

A partir de una etnografía multi-situada llevada a cabo durante 14 meses en el norte de Santiago, examino cómo la introducción de una serie de políticas de salud y de la agenda de salud mental global ha interactuado e impactado a los migrantes haitianos en el contexto del Chile neoliberal posdictadura (1990-2019). Específicamente, exploro las interacciones entre instituciones sociales y de salud, profesionales de salud mental, tecnologías psi y migrantes haitianos, destacando procesos de subjetivación y la vida cotidiana de los migrantes. Sostengo que los migrantes haitianos participan en procesos de subjetivación heterogéneos en sus interacciones con instituciones sociales y de salud, desafiando valores normativos de integración a la sociedad chilena. Estos procesos están marcados no sólo por la presencia, o exposición a, intervenciones psi y discursos sobre salud mental, sino también por el grado de compatibilidad entre un lenguaje psiquiátrico y neurológico y los ideales y marcos morales de los haitianos.

“I believe we can successfully treat Haitian patients at the psychiatric hospital and the health network,” said Pedro^1^ as we walked with another psychiatrist down one of the corridors after the monthly mental health consultancy at a family health care center (*centro de salud familiar* [CESFAM]).^2^ Both psychiatrists, unlike most of the psychologists and social workers who participated that day in the meeting, felt optimistic about the work with Haitians. They trusted the recent local health department (LHD) initiatives in “intercultural health” and the health teams’ capacity. However, in turn, Pedro also pointed out that the Ministry of Health and local health institutions should focus their efforts in improving Haitians’ level of access and adherence at primary health care centers and familiarize teams with Haitians’ lives and migratory conditions, Haitian-Creole medicine and Vodou. He added: “We knew a little bit about life in Haiti and the humanitarian crisis, but we didn’t know too much about health services and the role of healers and spirits (…). However, we have achieved some goals at the hospital, good outcomes.”

The arrival of Haitian migrants has triggered various frictions, conflicts, and challenges in health care centers in northern Santiago since the early 2010s.^3^ Psychologists, social workers, and other health practitioners at the CESFAM revealed various issues in their clinical and community encounters. They mainly stressed that Haitians did not “attend to psychological consultations” and did not “adhere to treatments.” Moreover, psychologists primarily reported several “unknown syndromes” and “strange etiologies” related to ancestors, spirits, and curses. In this scenario, clinical meetings and informal spaces such as lunch hours and corridor conversations became spaces where practitioners questioned their own professional competencies. They particularly emphasized that working with Haitian migrants within a precarious healthcare system led them to challenge their “intercultural approach,” their understanding of psychiatric diagnostic categories and treatments, and the concept of the mind itself.

In this article,^4^ I examine how the introduction of a series of health policies and the global mental health agenda has interacted with and impacted Haitian migrants in the context of a postdictatorship neoliberal Chile (1990–2019).^5^ Specifically, I explore the multiple interactions between health and social institutions, mental health practitioners, “psy” technologies, and Haitian migrants, highlighting migrants’ subjectivation processes and everyday life. By “psy” technologies, I refer to a set of techniques, practices, and discourses involving psychotherapies, psychological evaluation techniques, and pharmacological treatments, which potentially operate as mechanisms of power, normalization, and social control (Foucault 2008; Rose 1996; Rose and Abi-Rached 2013). I highlight how these technologies shape individuals’ representations about themselves, their afflictions, and well-being.

Rather than adopting a teleological position, I conceptualize the relationship between health institutions, psy technologies, and Haitian migrants as part of a broader and more complex social framework in which multiple life histories, social bonds, subjectivities, and experiences take form (Béhague 2016). I foreground migrants’ subjectivation processes and their individual and collective potentialities (Mezzadra and Neilson 2013) instead of focusing exclusively on biopower and aspects of governmentality associated with biomedical and psychiatric practices (Savransky 2014). By subjectivation, I describe a dynamic and ongoing process through which individuals come to understand themselves as subjects, as opposed to fixed or stable subjectivities (Biehl and Locke 2010; Deleuze and Guattari 1987). Therefore this work builds an exploration on subjective aspects, subjection, resistance, and resignification (Butler 1997) by emphasizing migrants’ interactions with biopsychiatry, magic, and religion not only in health care centers but also in cross-border family structures and various social institutions (e.g., churches and schools).

I argue that Haitian migrants engage with heterogeneous subjectivation processes in their interactions with health and social institutions, challenging normative values of integration into Chilean society. Many Haitians I worked with refused mental health interventions and managed their afflictions in family and religious spaces. These spaces provided them with social actors (e.g., extended family, pastors, healers) and moral frameworks through which they could share their lived experiences. Nevertheless, Haitians who were involved in psychiatric interventions in specialized centers (e.g., psychiatric hospital), as well as migrant children and adolescents who participated in mental health workshops in schools, engaged to a different extent with subjectivation processes anchored in neurobiological functioning. They shaped hybrid representations of themselves, and their afflictions and well-being based on neurobiology, Haitian-Creole medicine, and Vodou.

This article is based on a multi-sited ethnography conducted over 14 months in 2018 and 2019 in a low- to middle-income neighborhood in northern Santiago. I began observation sessions at the CESFAM with the largest number of migrants registered, according to the LHD. Following key actors and relationships, I conducted observations in specialized centers, the LHD, the North Metropolitan Health Service, migrants’ homes and everyday life spaces, and Catholic and Protestant churches and schools. I also conducted 45 semi-structured interviews with various participants, including health practitioners, migrants and their relatives, and other key stakeholders such as public policymakers, priests, pastors, and Vodou healers. Of the 21 interviews I conducted with Haitians, most were conducted in Spanish, with the exception of three which were carried out in French, and four in English. Additionally, a female research assistant conducted five interviews in Spanish with Haitian women, with the intention of enhancing communication and minimizing gender bias and potential conflicts with male relatives.

I begin by showing how health institutions and mental health practitioners faced a series of frictions, conflicts, and challenges by working with Haitian migrants at the CESFAM. I describe how institutions and practitioners dealt with Vodou and Haitian Creole-medicine and how they developed intercultural health initiatives in a public health system marked by precarity. Then, I describe how most mental health practitioners valued community-based interventions as a cost-effective strategy for working with Haitians in a precarious context. However, because of mental health policies and plans implemented since the 1990s, they carried out mainly individual interventions that pursued achieving health goals^6^ (*metas sanitarias*) framed in an economic rationale. Although most of them reflected critically on the ontological and epistemological aspects of biopsychiatry when working with migrants, in their clinical interventions they tended to translate the Haitians’ afflictions through a psychiatric and neurological language, thereby foreclosing alternative epistemological perspectives.

In the following sections, I describe three different Haitians’ subjectivation processes from their interactions with health and social institutions. Through these processes, I show how Haitians, to different extents, assimilate, hybridize, and refuse mental health interventions. Finally, I draw some conclusions about how interventions shaped migrant Haitians’ representations of themselves, their afflictions, and their well-being based on neurobiology, Haitian-Creole medicine and Vodou. I emphasize that this process is marked not only by the presence of, or exposure to, mental health discourses and interventions but also by the degree of compatibility between a psychiatric and neurological language and the Haitians’ ideals and moral frameworks.

## Haitian migration, Vodou, and “intercultural” initiatives in a precarious public health system

From the initial days of my fieldwork, I observed how CESFAM practitioners had progressively recognized various issues within mental health and sexual and reproductive programs when they worked with Haitian migrants at primary health level since 2010. Psychologists and social workers were particularly concerned about the low level of access and poor adherence to psychological consultations. According to them, Haitians tended not to attend the mental health “first consultation,” to which they were typically referred by a family doctor at the CESFAM. This tendency implied the “potential chronicity” of health conditions and “administrative issues,” leading to the “loss of clinical hours” and, consequently, noncompliance with “health goals.”

Moreover, practitioners were most concerned about specific experiences with Haitian patients. In clinical settings, some Haitians attributed afflictions and pain to bodily fluids, the effects of curses, as well as afflictions caused by messages from ancestors and spirits. Psychologists and social workers usually labeled these cases as “extreme” or “paradigmatic,” going on to say that the cause was not “coherent” or did not have an “easy solution.” They felt intrigued, conflicted, and frustrated when faced with these incommensurable encounters. Daniela, a CESFAM psychologist, defiantly said to her colleagues in the consultancy meeting mentioned above:

Do you truly think we will ever be able to do something like psychotherapy with Haitian patients? [with ironic tone] (…). I sincerely believe not! (…) At the psychiatric hospital, a psychiatrist says that a patient is psychotic, that he has schizophrenia … ok, so that means drugs and therapy (…), but then the [cultural] facilitator^7^ says that this is due to Vodou and that the patient must be left alone (…), that they aren’t hallucinations, that they are the voices of their ancestors or spirits and that they will pass in a couple of days… a kind of lucid psychosis … Who do we believe? (…). Vodou healers and pastors do more than we do in these cases.

Daniela’s words represented the mental health practitioners’ feelings of failure when they worked with Haitians. Furthermore, they also unveiled the active involvement of institutions and social actors that were previously unfamiliar, in this context, to practitioners, such as churches and pastors.

In this context, practitioners urgently request that the LHD organize training sessions on “intercultural health.” In 2013 and 2014, the LHD organized some talks with experts from universities and ONGs. However, it was not until 2015 that the LHD provided structured training sessions through the Migrant Program – a local initiative funded by the Ministry of Health over three years. Training sessions offered practitioners what they called an “intercultural perspective” for working with migrants. In 2018, the program prioritized training on “Haitian-Creole medicine”^8^ and “Vodou” based on practitioners’ reports of “extreme” and “paradigmatic” cases. To do this, the program invited Haitian experts in public health and medical anthropology who had engaged to varying extents with the public health system over the previous years.

Institutional initiatives, training sessions, health consultants, and practitioners’ clinical experiences with Haitians made Haitian-Creole medicine and Vodou, remarkably relevant within the CESFAM. Vodou is a religion that combines indigenous West African religions and Catholicism (Hurbon 1999). It situates individuals within a universe made up of ancestors, spirits, and the natural world (“lwa”) (Price-Mars 1990 [1928]). Vodou is not only a religion to which subjects may or may not ascribe but also one of the foundations that shapes religious, health, and legal practices of a large part of Haitian society (Ramsey 2011; Vonarx 2012). It is also a health system that interacts with biomedicine, local systems of healing, and other religions, shaping heterogeneous health trajectories (Damus and Vonarx 2019; Khoury et al. 2012).

Rather than reproducing a culturalist approach to Vodou (Farmer 2004; Trouillot 1995), consultants and practitioners engaged critically with historical, political, and contextual aspects of health and religion in Haiti. In training sessions, consultants avoided conveying cultural stereotypes about Vodou. A Haitian consultant with a medical anthropology background said in an interview:

We don’t want to convey an image of Vodou as something exotic (…). We want to teach that Vodou is something dynamic and changes depending on the context (…). For example, if you don’t have resources, if you don’t have an official biomedical health system, many aspects of Vodou will still be alive (… .). Vodou is the official health system for most Haitians.

The consultants’ critical approach resonated with mental health practitioners’ approach in historical and contingent terms. Practitioners’ structural-based mental health approach – predominant in Chile and other Latin American countries since the 1960s and 1970s (Abarca-Brown and Ortega 2024) – led them to primarily focus on contextual and socioeconomic aspects involved in Haitians’ health trajectories, rather than cultural and other structural factors (e.g., racism, stigma, among others). In addition, practitioners familiarized themselves with Haiti’s historical, political, and socioeconomic aspects through the media of the United Nations Stabilization Mission in Haiti. The mission aimed to establish a safe and stable environment that would promote strengthening institutions, the rule of law, and the protection of human rights from 2004 to 2017 (UN 2019). Television and newspapers periodically showed how the Chilean army and police developed several actions along with international troops. In this context, practitioners knew about their patients’ living conditions in Haiti, especially after the earthquake of 2010.

Training sessions allowed practitioners to familiarize themselves with the complex and dynamic interweavings between Vodou and the structural aspects involved. These led them to understand, among various topics, the reasons why Haitian-Creole medicine and Vodou became the “official health system,” the reasons why Haitians often used primary health care centers in case of emergencies rather than for prevention purposes, and the heterogeneity of practices involved in health trajectories. Nevertheless, while the Migrant Program assessed the training sessions’ impact as “positive,” some practitioners expressed concern about these sessions’ success. In mid-2018, Daniela said in a conversation:

It has been a very short time since we learned about the importance of Vodou among Haitians; we had no idea about their culture (…). Now we have a greater understanding of the hallucinations or trances … and the beliefs and rituals of pregnant women reported by the midwives (…) but it is very little, and these [training sessions] must reach all the teams and practitioners (…) there is a lack of resources in the health system. The Migrant Program cannot afford to have systematic training sessions every month.

As Daniela mentioned, the lack of resources played a crucial role in limiting the number of training sessions in different health centers. In 2018, sessions became less frequent, and only a few CESFAM practitioners had the chance to get involved in training.

Moreover, according to Daniela and her colleagues, precarious health system conditions did not set the conditions for a work environment where they could “reflect in intercultural terms.” As I observed at the CESFAM, these conditions included: poor public health infrastructure, scarcity of mental health practitioners, high demand for mental health services, a high number of consultations per day (approximately 12–14), reduced length of consultations (30 minutes), and a limited availability of Haitian cultural facilitators. In this scenario, practitioners reported that these conditions led them to “always do the same thing;” in other words, to standardize clinical interventions. María, a CESFAM psychologist who had been working in primary health care for more than 50 years, said:

The health system burns you out (…). Psychologists repeat the same structure of interventions during consultations (…). They assess patients’ symptoms during the last weeks and then give them some explanations and guidance. You can see up to 50 patients in a week. Thus, you do not have much time for in-depth psychotherapeutic work and much less time for reflective work in intercultural terms (…). Interculturality is sometimes just a nice discourse.

Although the Migrant Program initiatives led practitioners to reflect critically on the role of cultural and structural aspects involved in Haitian migrants’ health trajectories, working conditions did not allow practitioners to adopt an “intercultural perspective” in their practice.

## Psychiatric and neurological language, translation, and cultural de-substantialization

Mental health practitioners who usually identified themselves as involved in “community psychology” and “critical perspectives in psychology” dealt with this lack of resources by prioritizing community interventions as a cost-effective strategy. For them, psychologists and social workers had to reconceptualize and prioritize actions that they regularly carried out in primary health care. For instance, they highlighted actions such as home visits and community-oriented interventions in neighborhood councils, schools, and sports clubs, among others. Furthermore, based on their experience in the training sessions, this group emphasized the importance of working with Haitian families in their everyday spaces and the role of churches, priests, and pastors in Haitians’ health trajectories.

However, although most mental health practitioners recognized the value of community-based interventions, some did not endorse this strategy. On the contrary, they argued that transformations had to begin at a health policy level; otherwise, these community initiatives would depend on personal efforts. In an interview, a CESFAM psychologist told me:

We spend most of our time on individual interventions. We prioritize compliance with health goals. So, it is challenging to do community work as such. Community interventions are often psychoeducational interventions. They are not more than that. But they are registered as “community interventions.” This does not mean working “with” the community (…). There needs to be more time and resources for real community interventions.

His words revealed how practitioners dealt with the precarity of the public health system and their various understandings of mental health and community-based interventions, approaches deeply rooted in the specific historical and sociopolitical contexts of the country over the last 50 years.

The historical, political, and sociocultural rupture provoked by the coup d’état in 1973, the subsequent civic-military dictatorship (1973–1989), and the implementation of neoliberal policies since the 1980s play a crucial role in the origins of these different mental health approaches. The dictatorship erased community psychiatry initiatives developed during the 1960s and 1970s and impoverished public health services. After the end of the dictatorship, the state designed a mental health policy inspired by community psychiatry initiatives, aiming to restructure psychiatric care based on territories and communities (Araya et al. 2009). This policy was framed within an economic rationale, creating indicators such as “number of new patients registered,” “community interventions,” and “health goals” and linking them to funding availability. The policy materialized in three community-oriented mental health plans from 1993, 2000, and 2017. The second plan, for example, promoted actions such as the National Program for the Detection, Diagnosis, and Treatment of Depression in primary health care – the program was the first of its kind in a lower-middle-income country.

Health policies and plans have legitimated mental health programs and practitioners since the 1990s. Local studies conducted in Santiago have shown that the figure of the psychologist was barely known in the 1980s and early 1990s (Prado et al. 1988). In impoverished sectors, the image of the psychologist – disseminated primarily through the media – was that of a “doctor of the soul,” and the consulting population tended not to distinguish their role from those of other health professionals. People tended to consider the experience of receiving psychological help as something “unknown” and “culturally distant” (Winkler 1993). Mental health practitioners’ legitimacy gradually changed in the postdictatorial context. For instances, while there were no more than five psychology programs in the country in 1980, 40 universities offered 87 psychology programs in 2008, partially because of the privatization of the educational system (Urzúa 2008).

These policies and plans have provided Chilean society with a psychiatric and neurological language to speak about afflictions, suffering, and well-being in a postdictatorial context marked by an accelerated modernization process, the development of a liberal market economy, and an increase in socioeconomic inequalities (Aceituno et al. 2012). In other words, mental health institutions, actors, and practices have provided people with language compatible with ideals of responsibility and meritocracy, as well as with the privatization of suffering and loneliness (Han 2012). In this context, mental health consultations in primary care centers increased 14.3% between 2007 and 2016 (Minoletti et al. 2018); and mental health disorders are currently Chile’s leading cause of medical leave (28.7% of total leave) (Superintendencia de Seguridad Social 2020). Researchers have also revealed an increase in the prescription of psychotropic drugs over the last three decades (Aguilera and Arenas 2023).

Although the Migrant Program provided practitioners with a cultural frame to reflect critically on the ontological and epistemological aspects of biopsychiatry, they tended to translate Haitians’ afflictions into a psychiatric and neurological language, especially in individual consultations at the CESFAM. The use of psychiatric categories mainly allowed them to limit incommensurable experiences in clinical encounters by neglecting mainly ethnic and moral aspects involved in Haitians’ health trajectories. For instance, they translated Haitians’ communication with ancestors, messages from spirits in dreams, and bodily rigidity resulting from possessions, into psychiatric symptoms and categories such as “hallucinations,” “catatonia,” “psychotic episodes,” and “schizophrenia.” As I will describe in the following section, this language became useful not only for diagnosis but also for explaining how critical events and afflictions were associated with specific neurobiological functioning.

I have called this translation process cultural de-substantialization.^9^ This is the practice through which practitioners – after familiarizing themselves with a cultural frame in a public health context – omit or neglect cultural aspects from the therapeutic relationship so that they can successfully translate afflictions within the coordinates of contemporary biopsychiatry. By doing this, practitioners tended to decontextualize Haitian patients’ experiences, foreclose other epistemes, and evaluate cases as “extreme” or “paradigmatic.” Moreover, they occasionally racialized what they called “cultural differences” in clinical spaces with Haitian patients (e.g., “Afro-descendant people are more likely to have psychosis”) and situated them in a place of “insanity,” “abnormality,” “disruption,” and “transgression.”

Various researchers have recently focused on the role of translation and politics in psychiatry, mental health, and migration. They have examined the mechanisms of translation involved at institutional and clinical levels, highlighting the politics of recognition (Giordano 2014), the politics of visibility (Varma 2021), and the politics of humanitarianism (Fassin and Rechtman 2009; Ticktin 2011). Through the concept of cultural de-substantialization, I emphasize how the politics of “multicultural neoliberalism” (Bolados García 2012) prevent practitioners from adopting a cultural frame beyond a discursive level in everyday practice and thus shaping a “space of mediation” (Nathan 2015) for the (non)translation of otherness in public health contexts.^10^ This form of multiculturalism is not simply a reduced version of multiculturalism (Hale 2005) but rather an expansion of neoliberalism into previously overlooked sociocultural realms such as “intercultural health” in postdictatorial Chile. Therefore, this narrow adoption of a cultural frame leads practitioners to perpetuate the coordinates of biopsychiatry and limit the circulation of different epistemes in public health.

Practitioners’ approach to the mentioned “extreme” or “paradigmatic” cases illustrates the cultural de-substantialization process. For instance, María attended to a Haitian woman who, along with her husband, said that “a man” had been following them for a few days. The couple then clarified that rather than a man, it was a spirit guided by a Haitian man. This man wanted revenge since the couple refused to help him while he was looking for a job. From the psychologist’s viewpoint, the couple’s “hallucinations” were a “lucid psychosis” (*psicosis lúcida*). She argued:

It was strange for me because it was a couple with well-structured minds, and they both saw the same man (…). However, of course, on the other hand, I cannot think of another explanation other than psychosis.

After asking her about the diagnosis and the follow-up of this case, the psychologist replied:

I registered them in the system as having a psychotic break and referred them to the psychiatric hospital, but after three days, the couple’s visions ended (…). These patients did not become severe cases, so we forgot about them and moved on to the next patients. I would like to have said, “stop, let us analyze these cases,” but it is impossible with the high number of patients.

The prominence of psy and neuro languages and practices not only offered mental health practitioners some references to translate Haitians’ afflictions into a psychiatric and neurological language but also – as I will show in the following sections – delineated different subjectivation processes both in the first and second migrant generations.

## Becoming a (neuro)migrant: The assimilation of mental health interventions

Haitians did not typically seek psychological help. At the CESFAM, family doctors primarily referred Haitian women for a “psychological evaluation” when they identified somatic symptoms without an identifiable organic cause. Although patients initially agreed with family doctors to attend the psychological consultation, most did not attend or they canceled sessions in advance. Only a few came to the “first consultation” a couple of weeks later. Among them, I knew three Haitians-two middle-aged women and a young man – who attended the first session driven by mental health issues. They experienced depressive symptoms related to family separation and living conditions. They shared high-middle-class backgrounds in Haiti and the fact that they were familiarized with mental health services due to their access to psychological and social work interventions in countries such as the US and France.

Haitians who participated in the first consultation after the referral perceived their attendance as a necessary “obligation” or “procedure” they were required to fulfill. As I observed in the consultation room,^11^ patients mostly looked restless during the session. They usually said that they did not know the psychologist’s role or that the referral was not justified because they were not mad. In subsequent interviews, Haitian women added some issues related to privacy and gender. A woman said, “I went once to the psychologist, and she told me that I could speak whatever I wanted, but I didn’t know her (…). My husband said I should not waste my time on these things and should focus on home.”

Psychologists’ interactions with the few Haitian patients at the CESFAM were brief. Haitians often discontinued treatments after the first or second session. In these interventions, psychologists typically offered an explanation that connected critical events, afflictions, and neurobiological functioning. They frequently linked Haitians’ emotional conflicts resulting from family separation, unemployment, and housing conditions to “cerebral,” “neuronal,” and “neurotransmitter” functioning. For example, psychologists, often adopting a pedagogical tone, said to patients, “Since your house was broken into twice, it is normal to be afraid; that makes your adrenaline rise, and you become more paranoid;” “Being away from your family makes you anxious, and that makes your thoughts and brain not work very well;” or “If you relax, you will sleep well, and your brain connections will work better.”

These interventions shaped incipient migrant Haitians’ representations of themselves, their afflictions, and their well-being. Some Haitians revealed a process of assimilation of a psychiatric and neurological language, which interacted with Haitian-Creole medicine, religions and Vodou. Haitians diagnosed with disorders such as “psychotic episodes,” “schizophrenia,” and “bipolar disorder” – and who, consequently, were an object of a greater number of psychopharmacological and psychotherapeutic interventions – engaged mainly with this form of subjectivation.

The recent life of Paul, a 24-year-old Haitian man, illustrates how interventions can impact and shape Haitians’ subjectivities and everyday lives. Paul arrived in Santiago from Port-au-Prince, persuaded by his older brother, who was already in Chile, and encouraged him to study there. Paul arrived at the age of 21. Since then, he has worked in sectors such as construction and materials transportation. Some weeks Paul combined both activities, working up to 18 hours when truck unloading occurred at night. After living in Santiago for five months, Paul received the news that his grandfather had died because of the collapse of one of the house walls where he lived in Haiti. After this, Paul’s various manifestations revealed that “he was not well,” in his brother’s opinion. Insomnia, discouragement, and a constant feeling of unrest afflicted him. His brother also noticed that Paul “talked” with his deceased grandfather. After four days in this same state, police brought Paul to the emergency room at the psychiatric hospital in the middle of the night. According to his brother, Paul was “agitated” and “violent.” He added, “Paul was screaming and screaming. He grabbed a stick and defended himself against someone (…) he also called his grandfather. The neighbors woke up and called the police.”

Paul is a representative case of how Haitians – especially men – were admitted to tertiary care. I had the opportunity to meet him eight months after the hospitalization while he was accompanying his brother’s wife for a health check at the CESFAM. Although Paul vaguely remembered his time at the hospital, he told me he was there for approximately two weeks. A couple of months later, his brother told me that Paul was diagnosed with “schizophrenia” and that he had to attend medical checkups and “take some pills in the morning and at night.” His brother took an active role in his pharmacological treatment, reminding him to take it daily. He pointed out, “The doctor told us that when you miss your country and life there, it can affect your brain (…). They said [Paul] had to keep taking his pills, and he did that until they ran out.” Nevertheless, Paul did not attend the checkups alluding to work reasons.

Paul engaged with incipient representations about his affliction based on neurological functioning, revealing how mental health interventions impacted and provided him with a psychiatric and neurological language. At his home, Paul told me:

The doctors taught me that my head, my brain, sometimes does that [psychotic break with psychomotor agitation]. I don’t think straight (…) When this happens to me, my brain switches off, and I don’t talk with anybody, and I feel nothing, without any thoughts (…). The doctor said I should do activities like playing football or going to church.

These brain-based representations interweaved with other epistemes. Although Paul and his family did not identify themselves as Vodouists but as Protestants,^12^ their representations of personhood, body, and afflictions and their conceptualizations about the etiology of health conditions and healing practices were rooted in Haitian-Creole medicine, Protestantism, and Vodou. The same day, Paul said:

My brother told me that maybe God was angry because we were not living in Haiti with our family (…). But the doctor said that if I am healthy, God and my body can protect me from a curse (…). I speak with my grandfather always (…). He is dead. He is not here, but sometimes I feel him here (…). The doctor told me that talking with my grandfather is good for me, but it is not good if I can hear him shouting at me (…). He told me that if my grandfather shouts at me and I feel scared, I have to go to the hospital because my body is sick.

Paul presented a significant health improvement after the hospitalization. He returned to work and began to regularly attend a Methodist church. Paul said, “I feel calm in the church (…). I feel connected with my family in Haiti.” I visited Paul at his home two months later – eleven months after his crisis. He was retaking his drugs. A social worker visited and gave him an appointment with the psychiatrist for a follow-up. She identified “high levels of anxiety” and “paranoid anxiety.” In fact, Paul confirmed this by saying that he felt “unwell” [“no bien”]. Moreover, he said to me that his brother occasionally prepared him an herb decoction “for the peace of his soul,”^13^ recommended by the Haitian pastor at the church. Regarding the appointment, Paul said: “the doctor said that I must take my pills because if my brain is not well, I am going to feel restive [*no tranquilo*] (…). And he said that it is fine to drink the medicine of my brother as well.”

The practitioners’ approach at the hospital played a crucial role in the assimilation/hybridization of different epistemes. Pedro, who was one of the psychiatrists who attended Paul, highlighted how practitioners at the psychiatric hospital could “dedicate more time to cases,” unlike practitioners at the CESFAM. He stressed that, although practitioners there do not have “unlimited resources,” they can “think together about clinical cases and find solutions with social workers and occupational therapists.” However, in turn, he recognized that there was no “institutional approach or guidelines for working with migrants.” Instead, practitioners’ approaches depended on individual professional backgrounds. In an interview, he pointed out, “I identified myself as close to the French school of psychoanalysis, so I am close to ethnopsychiatry and Devereux and Nathan. But I think that only recently we are taking these authors seriously because of the new multicultural context.” Regarding Paul, he pointed out:

For me, he is stable as he takes his pills and has family support; those are the main reasons for his better condition (…). But I think it is essential to have an open mind and be receptive when patients like Haitians or others bring with them religious and magical contents (…). They can symbolize their conflicts and manage their angst (… .). If he wants to drink a decoction that people do in Haiti, it is fine. But we need to know more about these kinds of practices.

Practitioners’ approaches, such as Pedro’s, shaped a space where different epistemes flow together complexly. The prominence of biopsychiatry in a precarious public health service foreclosed other epistemes – as different researchers have shown similarly in different contexts (Duncan 2014; Giordano 2014). Nevertheless, Pedro and other practitioners provided a space where, along with the use of traditional diagnoses and psychotropic drugs, a certain degree of mediation took form through which patients like Paul could talk about themselves and their afflictions. *La escucha psicoanalítica* (psychoanalytic listening) – as psychiatrists and psychologists at the psychiatric hospital called it – became a therapeutic tool itself through which they could “give a space to Haitians,” contest the biopsychiatric episteme and challenge public mental health policies at room consultations (Brotherton 2020). Thus, la escucha psicoanalítica opened a space in public health where Haitians engaged with subjectivation processes through which they assimilated a psychiatric and neurological language and hybridized this with Haitian-Creole medicine, religion, and Vodou.

## Becoming a migrant: The refusal of mental health interventions

The Haitians I worked with tended to face their afflictions in spaces other than the public health system. They engaged with heterogeneous health and religious practices. Various researchers have extensively described in a similar way how Haitians manage their afflictions in family and community spaces, churches, and with Vodou healers (Khoury et al. 2012; Vonarx 2012). They have also shown how religious spaces have provided conditions for the strengthening of family, community, and national ties in the Haitian diaspora (Brown 1991; Rey and Stepick 2013). However, what I could note in my fieldwork was that a few CESFAM practitioners – especially family doctors – played a role in these health trajectories by underestimating religious spaces, actors, including pastors and healers, and moral frameworks involved.

Cultural facilitators were critical witnesses to how these practitioners dismissed religious spaces and epistemes and, in turn, how Haitians refused mental health interventions. In an interview, a facilitator said:

There are doctors who say that healers and pastors will not help them. They say that patients must take their pills for depression (…). This is the way how people get well, not at the church, they say. And people feel confused and sometimes angry with this (…). What doctors say here is sometimes difficult to understand for patients (…) Sometimes I don’t understand (…); there are technical words that don’t exist in Creole (…). They say things about the brain, hormones, and neurotransmitters (…). I don’t want you to take what I am going to say badly, but I don’t know if what psychologists do here is helpful for the people of my country (…). Haitians don’t believe in mental health problems like people in Chile. If someone feels bad, that person will go to church or a healer.

Facilitators’ words revealed some mistrust regarding the interventions’ success based on the gap between these practitioners’ approach and Haitians’ religious and healing practices.

Lía is a typical case of how Haitians refuse mental health interventions and instead engage in health and religious practices. Lía arrived in Chile in 2015 when she was 21 years old. She lived with her sister and brother-in-law in a shared room. Lía divided her time between working in a grocery store and participating in activities with her family group. She enjoyed walking around squares and parks and staying at home “studying the Bible.” Lía participated in a Protestant church near her home, where she achieved a certain degree of recognition. In some services, the pastor used her as an example to other worshipers, stating that she was a “true daughter of God” and was “one of the people whom Chile needed.”

In 2018, Lía experienced headaches, back pain, and lack of energy. Lía’s brother-in-law attributed her afflictions to being “older” and not having found “a man for having her own home.” Following her sister’s recommendations, Lía attended an appointment with a family doctor at the CESFAM. There, she attributed her afflictions to the overload of work and the lack of time for attending church. She told me, “The doctor referred me to the psychologist. He said that maybe I had depression and needed psychological treatment rather than a church.” Lía attended the first psychological consultation. However, after the psychologist explained the purpose of the session, Lía told me that at that moment, she replied, “I do not need a psychologist. What I need is God (…) God is the one who orders my life and my family.” After that session, Lía did not attend another consultation.

Patients like Lía underscore how moral frameworks, marked by gender and intergenerational dynamics, delineate the interactions between Haitians and health and religious institutions and actors. For instance, some CESFAM practitioners considered that Haitian women’s “hermeticism” and “conservatism” became “obstacles” to facing their afflictions. Attempting to encourage “autonomy” and “independence” in their interventions, practitioners conceptualized women as “submissive” and “passive.” This Haitian-otherness construction had implications in cases in which interventions sought women’s “empowerment” to achieve “greater independence” or “autonomy” – for example, in cases of domestic violence or contraception (Abarca-Brown 2023).

## The intergenerational issue: Psy technologies and Haitian children

Mental health interventions found a positive reception when practitioners worked with children and adolescents in neighborhood schools. CESFAM psychologists and social workers regularly carried out prevention activities such as talks and workshops in those spaces. They talked about “psychological” and “psychosocial” issues such as “depression,” “suicide,” and “bullying” because school communities considered them relevant topics. Practitioners explained to professionals and students how life events could impact them in cognitive, emotional, and behavioral terms and affect their neurobiological development. They talked in a familiar and pedagogical tone, inviting them to participate and express their concerns and afflictions during workshops or in more intimate spaces with teachers and educational psychologists. Adolescents specially used these spaces and reproduced a psychiatric and neurological language provided by those professionals. In the case of migrant students, those who had lived longer than five years in Chile tended to internalize this language.

In this scenario, the interventions carried out by psychologists and social workers in schools triggered various tensions and conflicts between Haitian parents and health and education institutions, as well as within Haitian families between parents and children. Parents often contested and refused mental health practices recommended by teachers and practitioners. They demanded explanations regarding why their children were referred to psychological consultations at the CESFAM and diagnosed with conditions like “behavioral problems” and “ADHD.” Simultaneously, parents occasionally reproached their children, particularly adolescents, for seeking psychological consultations. At times, fathers held mothers accountable for their children’s conditions.

Stevenson’s recent life typically illustrates these frictions and conflicts. Stevenson was nine years old when he migrated with his parents in 2014. Upon arriving in the country, Stevenson entered the fifth grade. He adapted to the new school environment faster than other Haitian children, as he learned to speak Spanish during the first four months. Teachers highlighted his “good disposition,” “communication skills,” and ability to “adjust to the new rules” of the school. In 2018, Stevenson began the eighth grade, the Chilean school system’s last year of primary education. The school’s psychologist, after talking with the teacher in charge, referred Stevenson for a psychological consultation. Both professionals said Stevenson presented with a “poor performance” and “emotional and behavioral conflicts.” His teacher was concerned that Stevenson had slowed his performance in various subjects and showed signs of “irritability,” “impulsiveness,” and “aggression” with his classmates.

I had the opportunity to meet Stevenson when he attended his fifth psychological consultation with his mother at the CESFAM in 2018. While the psychologist recognized Stevenson’s progress in therapy, she criticized the school’s professionals arguing that they were not aware how the dynamics of racism had been affected Stevenson. She highlighted that teachers tended to overlook “micro racist practice” and “racialization dynamics.” Teachers ignored, she said, how the reproduction of such dynamics within the peer group affected migrant students. In her opinion:

Teachers and school psychologists sometimes don’t have much time to observe what is happening with children (…). Stevenson and his friends are becoming adolescents, and often, power dynamics begin within groups in which one tries to become a leader and surpass the others (…). Stevenson thought that being black made him poor or that he should return to his country. He has heard that from his peers. Children reproduce things they hear at home or on television.

Although the psychologist informed Stevenson’s mother of this situation, neither she nor her husband fully understood why their son should attend consultations. This situation created conflicts between Stevenson’s parents and the care system. According to Stevenson’s mother, her husband was “upset” with the referral arguing that his son was not “mad.” Stevenson’s father insisted on his wife not sharing details with a stranger. In addition, he was concerned that the sessions might “drive him mad.”

Stevenson valued psychological support spaces, unlike his parents. He told me that it was not only him who attended consultations but also other schoolmates. Indeed, Stevenson knew the diagnoses of his colleagues and the names of the psychologists at the center with whom they consulted. For Stevenson, sharing diagnoses and experiences in mental health was “normal” and something “everyone did at school,” becoming a mechanism of integration into his peer group. Although Stevenson knew his parents’ position regarding attending consultations, he said teachers sent him “to feel better.” In his words, “I come to talk, they make me draw; that is what the school psychologist told us. This is so that you feel good (…) and you do better in school.”

## Discussion and conclusion

I have explored the multiple interactions between health institutions, mental health practitioners, “psy” technologies, and Haitian migrants, highlighting migrants’ subjectivation processes and everyday lives. I have argued that Haitian migrants engaged with heterogeneous subjectivation processes in their interactions with health and social institutions, challenging normative values of integration into Chilean society. By foregrounding Haitians’ subjectivities and individual and collective potentialities, I have described how they engaged with at least three different subjectivation processes characterized by the assimilation and refusal of mental health interventions and how children become involved with them.

Health and social institutions have undergone a transformation within this emerging multicultural context. In health spaces, although practitioners have made significant efforts by adopting a cultural frame in health practices, factors such as health policies framed in an economic rationale, a precarious health context, and the predominance of biopsychiatry have limited the circulation of different epistemes. In parallel, churches have also become multicultural spaces. The arrival of migrants has led pastors to embody contradictions between adopting a moral and antimigration agenda associated with right-wing political sectors and legitimizing their work vis-a-vis migrants.

Haitians’ subjectivation processes allow us to question how health institutions and psy technologies are “making up” (Hacking 1985) Haitian patients based on representations of the human mind, behavior, and mental disorders anchored in neurobiological functioning. In other words, by reproducing a psychiatric and neurological language in health care centers and schools, mental health practitioners have provided Haitians with a neuro-based understanding of themselves, their afflictions, and their well-being. However, Haitians’ subjectivation processes reveal how the assimilation, hybridization, or refusal of a psychiatric and neurological language is related not only to the biopolitical and governmental action of health institutions, actors, and practices (Rose and Abi-Rached 2013) but also to how they embody frictions and contradictions between their moral frameworks and ideals and values (Ehrenberg 2018) promoted in different spaces in Chile. This work demonstrates how values and ideals related to family, gender, privacy, and intergenerational relationships become crucial in their interactions with mental health institutions and practitioners.

Finally, Haitians’ subjectivation processes underline the need to decolonize Western conceptualizations of the mind, especially in transcultural settings. Various contributions to the “theory of mind” could help us understand how various mental experiences reveal specific relationships between the mind, body, reality, and society (Descombes 2010; Hollan 2000; Luhrmann 2020). For example, focusing on how societies provide frameworks through which individuals perceive and interpret their mental processes (Luhrmann 2020) allows a more accurate approach to clinical phenomena – “trances,” “visions,” and “messages from ancestors,” among others – usually considered abnormal or pathological both in Haitian and other communities.

## Notes

1. Names and sensitive information have been modified to protect participants’ anonymity.

2. Primary health care level is primarily made up of CESFAMs. Practitioners, including family doctors, dentists, nurses, midwifes, nutritionists, psychologists, and social workers, work in CESFAMs. Generally, neighborhoods have between one and five CESFAMs, depending on their population size.

3. According to LHD documents, the number of Haitians increased fivefold in the neighborhood between 2015 and 2018 (around 5000 Haitians in 2019). This neighborhood became an attractive place to them because of its low housing prices, connectivity with commercial areas, and availability of religious centers.

4. This article is based on a broader project that sought to investigate how new discourses and practices related to migration, multiculturalism, and mental health have been shaped in postdictatorship neoliberal Chile.

5. I define the postdictatorial period as the period from the end of the civic-military dictatorship (1973–1989) to the *estallido social* (social outburst) of 2019. The estallido was a series of demonstrations and riots across the country between October 2019 and March 2020 in response to the cost of living, privatizations, probity crisis, and socioeconomic inequalities. In this context, international and national organizations denounced various human rights violations, including torture, sexual abuse, and rape.

6. The law 19.813 established an evaluation system called *metas sanitarias* (health goals). Monetary incentives are provided for health practitioners, based on the collective performance of the centers.

7. In 2017, the LHD hired Haitian cultural facilitators for translation and mediation in the clinical setting. Each CESFAM had at least one cultural facilitator. They were mainly psychologists and teachers. They usually worked with patients who had been living in Chile for less than two years and who were not yet sufficiently proficient in the Spanish language.

8. Haitian Creole medicine is a set of knowledge, practices, and values related to health and disease in which practices from biomedicine, herbal medicine, and Vodou, among others, converge (Damus and Vonarx 2019).

9. Following Hegel’s philosophy, I understand “substantialization” as the externalization of thought into concepts that impact on reality. Through this concept, I emphasize the process of “naming,” in this case, the notion of “culture” and its impacts in the transference relationship.

10. Here I am following Cristiana Giordano’s idea that “otherness lies in that which remains untranslatable” (2014: 16).

11. At the CESFAM, I conducted observations in the first practitioners’ interviews with Haitian patients.

12. In 2010, 86.9% of the population ascribed to Christianity. Within this group, 56.8% of Haitians declared themselves Catholic and 26.9% Protestant (Pew Research Center 2020). Although the same research center maintains that 2.2% of the population subscribes to folk religions, this data may be inaccurate because of the stigmatization of Vodou.

13. Although Paul did not remember the specific type of decoction, the pastor told me later that it was probably “Flor de Santa Lucía” (*Commelina elegans*) or “Caimitillo” (*Chrysophyllum oliviforme*).
